# Clusters of Human Infection and Human-to-Human Transmission of Avian Influenza A(H7N9) Virus, 2013–2017

**DOI:** 10.3201/eid2402.171565

**Published:** 2018-02

**Authors:** Lei Zhou, Enfu Chen, Changjun Bao, Nijuan Xiang, Jiabing Wu, Shengen Wu, Jian Shi, Xianjun Wang, Yaxu Zheng, Yi Zhang, Ruiqi Ren, Carolyn M. Greene, Fiona Havers, A. Danielle Iuliano, Ying Song, Chao Li, Tao Chen, Yali Wang, Dan Li, Daxin Ni, Yanping Zhang, Zijian Feng, Timothy M. Uyeki, Qun Li

**Affiliations:** Public Health Emergency Center of the Chinese Center for Disease Control and Prevention, Beijing, China (L. Zhou, N. Xiang, R. Ren, Y. Wang, D. Li, D. Ni, Y. Zhang, Z. Feng, Q. Li);; Zhejiang Center for Disease Control and Prevention, Hangzhou, China (E. Chen);; Jiangsu Center for Disease Control and Prevention, Nanjing, China (C. Bao);; Anhui Center for Disease Control and Prevention, Hefei, China (J. Wu);; Fujian Center for Disease Control and Prevention, Fuzhou, China (S. Wu);; Hebei Center for Disease Control and Prevention, Shijiazhuang, China (J. Shi);; Shandong Center for Disease Control and Prevention, Jinan, China (X. Wang);; Shanghai Center for Disease Control and Prevention, Shanghai, China (Y. Zheng);; Shaanxi Center for Disease Control and Prevention, Xi’an, China (Y. Zhang);; US Centers for Disease Control and Prevention, Atlanta, Georgia, USA (C.M. Greene, F. Havers, A.D. Iuliano, Y. Song, T.M. Uyeki);; National Influenza Center of the Chinese Center for Disease Control and Prevention, Beijing (T. Chen);; Chinese Center for Disease Control and Prevention, Beijing (Z. Feng)

**Keywords:** Avian influenza A(H7N9) virus, human infections, clusters, human-to-human transmission, China, viruses

## Abstract

To detect changes in human-to-human transmission of influenza A(H7N9) virus, we analyzed characteristics of 40 clusters of case-patients during 5 epidemics in China in 2013–2017. Similarities in number and size of clusters and proportion of clusters with probable human-to-human transmission across all epidemics suggest no change in human-to-human transmission risk.

Since December 2016, the number of human infections with avian influenza A(H7N9) virus in China has increased markedly (*1*,*2*), prompting concerns of pandemic influenza. Early signals of greater human-to-human transmissibility might be increased number and size of clusters of epidemiologically linked human infections and clusters of case-patients who are not blood relatives or increased numbers of case-patients with mild illness (*3*). To elucidate whether the increase in human infections during the fifth epidemic (2016–2017) in China was associated with increased human-to-human transmissibility of A(H7N9) virus, we compared the characteristics of clusters of A(H7N9) case-patients during the fifth epidemic with those of clusters of case-patients identified from the previous 4 epidemics (2013–2016).

## The Study

For each laboratory-confirmed A(H7N9) virus infection reported in mainland China, the provincial or local Center for Disease Control and Prevention (CDC) initiated a field investigation to monitor close contacts for illness signs and symptoms for 10 days after the last known exposure to a symptomatic index case-patient (*3*). Upper respiratory specimens collected from close contacts with respiratory symptoms were tested for A(H7N9) virus as previously described (*3*). Detailed information (e.g., demographic data, household and family relationships, exposures to index case-patients and poultry, and clinical management and outcomes) was collected from case-patients, close contacts with laboratory-confirmed A(H7N9) virus infection, and their close contacts. Collection and analyses of data from case-patients with influenza A(H7N9) virus infection were part of an ongoing public health investigation of emerging outbreaks and were exempt from institutional review board assessment in China (*2*).

An epidemic period was defined as September 1 through August 31 of the following year. A cluster was defined as >2 epidemiologically linked case-patients with laboratory-confirmed A(H7N9) virus infection and illness onset within 10 days of each other. Within clusters, probable human-to-human transmission was defined as occurrence of secondary infection in a person who had close contact with a symptomatic index case-patient but no known poultry exposure; possible human-to-human transmission was defined as occurrence of secondary infection in a person who had close contact with a symptomatic index case-patient and known poultry exposure.

We performed descriptive analyses of laboratory-confirmed A(H7N9) case-patient data reported to the China CDC during February 1, 2013–June 30, 2017. We described the number of clusters per epidemic period and compared cluster patient characteristics from the fifth epidemic with those from previous epidemics; specifically, we compared numbers of clusters; case-patients per cluster; and case-patient age, sex, underlying medical conditions, hospitalization, oseltamivir treatment, intensive care unit admission, invasive mechanical ventilation, and deaths. We compared the characteristics of sporadic (noncluster) case-patients with those of index and secondary case-patients; in clusters with probable human-to-human transmission, we also compared index with secondary case-patients. Because of our interest in human-to-human transmission, we focused our analyses on clusters in which secondary case-patients reported no exposure to poultry. We compared categorical variables by using χ^2^ or Fisher exact tests and median ages by using the rank-sum Wilcoxon test. All tests of statistical significance were 2-sided with a cutoff of p = 0.05.

As of June 30, 2017, we identified 84 infections comprising 40 clusters among 1,524 A(H7N9) case-patients reported since February 2013 (Table). Most clusters were located in southern and eastern China (Technical Appendix Figure). We identified 14 clusters in the fifth epidemic, compared with 4–11 clusters in prior epidemics. The 14 clusters in the fifth epidemic comprised 29 human infections; previous epidemics had clusters of 8–23 human infections per epidemic. In the fifth epidemic, 13 (93%) clusters had 2 infections each and 1 had 3 infections, compared with 23 (88%) clusters with 2 infections each and 3 clusters with 3 infections each among 26 clusters in epidemics 1–4. The proportion of all infections that occurred in clusters differed significantly among all clusters (p = 0.023) and was lowest during the fifth epidemic (4%).

**Table:** Features of sporadic and clusters of human infections with avian influenza A(H7N9) virus, mainland China, February 1, 2013–June 30, 2017*

Category	Total	Epidemic†				
		First	Second	Third	Fourth	Fifth
Overall infections						
Infections, no.	1,524	134	304	219	118	749
Deaths, no. (%)	599 (39.3)	44 (32.8)	126 (41.4)	99 (45.2)	47 (39.8)	283 (37.8)
Sporadic infections‡						
Infections, no.	1,440	126	281	207	106	720
Deaths, no. (%)	570 (39.6)	39 (31.0)	120 (42.7)	94 (45.4)	45 (42.5)	272 (37.8)
Cluster infections						
Infections, no. (%)	84 (5.5)	8 (6.0)	23 (7.6)	12 (5.5)	12 (10.2)	29 (3.9)
Deaths, no. (%)	29 (34.5)	5 (62.5)	6 (26.1)	5 (41.7)	2 (16.7)	11 (37.9)
All clusters						
Clusters, no.	40	4	11	6	5	14
Clusters with 2 infections, no.	36	4	10	6	3	13
Clusters with 3 infections, no.	4	0	1	0	2	1
Deaths of index case-patients, no. (%)	19 (46.3)	3 (75.0)	5 (45.5)	2 (33.3)	1 (16.7)	8 (57.1)
Deaths of secondary case-patients, no. (%)	10 (23.3)	2 (50.0)	1 (8.3)	3 (50.0)	1 (16.7)	3 (20.0)
Clusters with probable human-to-human transmission only						
Clusters, no. (%)	11 (30.0)	2 (50.0)	3 (27.3)	2 (33.3)	1 (20.0)	3 (21.4)
Clusters with 2 infections, no.	11	2	3	2	1	3
Clusters with 3 infections, no.	0	0	0	0	0	0
Infections, no.	22	4	6	4	2	6
Index, no.	11	2	3	2	1	3
Secondary, no.	11	2	3	2	1	3
Blood-related family members	12	4	2	2	2	2
Unrelated persons	10	0	4	2	0	4
Overall deaths, no. (%)	12 (54.5)	3 (75.0)	3 (50.0)	2 (50.0)	1 (50.0)	3 (50.0)
Of index case-patients, no. (%)	9 (81.8)	2 (100.0)	2 (66.7)	1 (50.0)	1 (100.0)	3 (100.0)
Of secondary case-patients, no. (%)	3 (27.3)	1 (50.0)	1 (33.3)	1 (50.0)	0	0

*Categorical variables among the 5 epidemics were compared by using χ^2^ or Fisher exact tests. Median age was compared by Wilcoxon test. The proportion of infections in clusters was lowest for the fifth epidemic; the proportion of infections in clusters per epidemic differed significantly during 2013–2017 (χ^2^ = 11.30; p = 0.023). The remaining items did not differ significantly among the 5 epidemics. The reporting of deaths may be delayed; therefore, these numbers may change as additional deaths are confirmed. †The first epidemic was defined as February through August 31, 2013; each subsequent epidemic was defined as September 1 through August 31 of the following year. ‡Excludes human infections that were identified in clusters.

Among the 40 clusters for all 5 epidemics, we classified 14 (35%) as probable and 26 (65%) as possible human-to-human transmission. The proportion of clusters with probable human-to-human transmission only did not differ significantly by epidemic (p = 0.842) (online Technical Appendix Table). A cluster of 3 infections in the fifth epidemic had possible and probable human-to-human transmission; 2 similar clusters were identified during the fourth epidemic. We identified no cluster with potential spread beyond 2 generations.

Among 14 secondary infections with probable human-to-human transmission during 2013–2017, we linked 4 to household exposures and 10 to exposures in healthcare settings, including 4 during the fifth epidemic. These 10 nosocomial infections included 3 blood relatives exposed to index case-patients (1 each in the second, fourth, and fifth epidemics); 1 unrelated household member exposed to an index case-patient in the second epidemic; and 6 unrelated patients exposed to index case-patients (1 each in the second, third, and fourth epidemics and 3 in the fifth epidemic).

The case-fatality proportion for all clusters combined was 35% (29/84), similar to that for all sporadic infections (40%) (Table). The case-fatality proportion for all clusters for the fifth epidemic was 38% and did not differ significantly for epidemics 1–4 (p = 0.23) (Table). The case-fatality proportion for patients with index and secondary infections in probable clusters not significantly different for the fifth epidemic (p = 0.84) or compared with previous epidemics (p = 0.53). Among all epidemics, the proportion of index case-patients in probable clusters admitted to an intensive care unit was higher than that for case-patients with sporadic infections (87% vs. 56%; p = 0.018), although this difference was not significant when data were limited to the fifth epidemic (Technical Appendix Table).

For clusters with probable human-to-human transmission, we found no significant differences between index case-patients and patients with secondary infections during the fifth epidemic or during each previous epidemic by median age, sex, underlying medical conditions, proportion hospitalized, proportion who received mechanical ventilation, or oseltamivir treatment (Technical Appendix Table). However, when we aggregated and analyzed data for probable clusters for all 5 epidemics, index case-patients were significantly more likely than patients with secondary infections to have received mechanical ventilation (60% vs. 14%; p = 0.02) and index case-patients were more likely than patients with secondary infections to be male (93% vs. 57%; p = 0.04) (Technical Appendix Table).

## Conclusions

Despite the surge in human infections with A(H7N9) virus during the fifth epidemic in China, the similarity in number and size of clusters and proportions of clusters with probable human-to-human transmission during 2013–2017 suggest no change in human-to-human A(H7N9) virus transmission risk over time. These findings suggest that the increase in human infections during the fifth epidemic probably reflects an increase in sporadic poultry-to-human A(H7N9) virus transmission over a wide geographic area in China (*1*).

 Although we restricted the assessment of human-to-human A(H7N9) virus transmission in probable clusters to secondary case-patients without identified poultry exposure, we may have overestimated human-to-human transmission in clusters if not all poultry exposures were identified and reported. We could have underestimated human-to-human transmission by excluding infections in possible clusters with exposures to both poultry and symptomatic case-patients. Only symptomatic close contacts of index case-patients were tested, possibly underestimating the size of clusters of patients with asymptomatic infections (*4*).

Clusters of probable limited human-to-human A(H7N9) virus infections, including in healthcare settings, underscore the value of adhering to recommended infection prevention and control measures to prevent nosocomial A(H7N9) virus transmission (*5**–**8*). Ongoing assessment of the epidemiology of human infections with avian influenza A(H7N9) virus to identify any increase in human-to-human transmission will inform pandemic risk assessment, preparedness, and response (*9*).

Technical Appendix Comparison of illness severity in clusters of human infections with avian influenza A(H7N9) virus and locations of clusters, mainland China, February 1, 2013–June 30, 2017.
