# Supplementary material for: Clusters of Human Infection and Human-to-Human Transmission of Avian Influenza A(H7N9) Virus, 2013–2017
Source: Emerg Infect Dis. 2018 Feb;24(2):397–400. doi: 10.3201/eid2402.171565 (PMC5782887; doi:10.3201/eid2402.171565)
Supplement: Technical Appendix — Comparison of illness severity in clusters of human infections with avian influenza A(H7N9) virus and locations of clusters, mainland China, February 1, 2013–June 30, 2017. [file 17-1565-Techapp-s1.pdf]

Article DOI: http://dx.doi.org/10.3201/eid2402.171565

Clusters of Human Infection and Human-to-Human Transmission of Influenza A(H7N9) Virus, China, 2013–2017

Technical Appendix

Technical Appendix Table. Comparison of illness severity in clusters of human infections with avian influenza A(H7N9) virus in mainland China, February 1, 2013 - June 30, 2017*

		Comparison of sporadic and cluster-associated index infections			Comparison of cluster-associated index and secondary infections with probable human-to-human transmission		
Characteristics		Sporadic infections	Index infections	*P* value	Index infections	Secondary infections†	*P* value
Total No. of infections	First epidemic	126	2	–	2	2	–
	Second epidemic	281	3	–	3	3	–
	Third epidemic	207	2	–	2	2	–
	Fourth epidemic	106	4	–	4	3	–
	Fifth epidemic	720	4	–	4	4	–
	Total	1,440	15	–	15	14	–
Median age (range), years	First epidemic	62 (2–91)	74 (61–87)	0.257	74 (61–87)	51 (33–68)	0.699
	Second epidemic	58 (2–88)	57 (57–60)	0.955	57 (57–60)	33 (30–70)	0.658
	Third epidemic	56 (1–88)	54 (48–59)	0.796	54 (48–59)	29 (1–57)	0.699
	Fourth epidemic	58 (14–91)	28 (13–35)	**0.001**	28 (13–35)	68 (21–87)	0.377
	Fifth epidemic	57 (3–92)	64 (29–66)	0.772	64 (29–66)	62 (39–63)	0.655
	Total	57 (1–92)	57 (13–87)	0.321	57 (13–87)	60 (1–87)	0.631
Male, no. (%)	First epidemic	88 (70)	2 (100)	1.000	1 (100)	1 (50)	1.000
	Second epidemic	198 (70)	3 (100)	0.558	3 (100)	3 (100)	–

Characteristics		Comparison of sporadic and cluster-associated index infections			Comparison of cluster-associated index and secondary infections with probable human-to-human transmission		
		Sporadic infections	Index infections	*P* value	Index infections	Secondary infections†	*P* value
Total No. of infections	First epidemic	126	2	–	2	2	–
	Third epidemic	142 (69)	2 (100)	1.000	2 (100)	2 (100)	–
	Fourth epidemic	71 (67)	3 (75)	1.000	3 (75)	0 (0)	0.143
	Fifth epidemic	515 (71)	4 (100)	0.582	4 (100)	2 (50)	0.429
	Total	1,014 (70)	14 (93)	0.082	14 (93)	8 (57)	**0.035**
Underlying medical conditions, no. (%)	First epidemic	41/125 (33)	2 (100)	0.113	2 (100)	1 (50)	1.000
	Second epidemic	115/281 (41)	2 (67)	0.571	2 (67)	1 (33)	1.000
	Third epidemic	79/207 (38)	1 (50)	1.000	1 (50)	1 (50)	1.000
	Fourth epidemic	51/106 (48)	1 (25)	0.620	1 (25)	2 (40)	1.000
	Fifth epidemic	308/700 (44)	2 (50)	1.000	2 (50)	2 (50)	1.000
	Total	594/1,419 (42)	8 (53)	0.371	8 (53)	7 (44)	0.715
Deaths, no. (%)	First epidemic	39 (31)	2 (100)	0.101	2 (100)	1 (50)	1.000
	Second epidemic	120 (43)	2 (67)	0.578	2 (67)	1 (33)	1.000
	Third epidemic	94 (45)	1 (50)	1.000	1 (50)	1 (50)	1.000
	Fourth epidemic	45 (42)	1 (25)	0.639	1 (25)	1 (33)	1.000
	Fifth epidemic	272 (38)	3 (75)	0.156	3 (75)	1 (25)	0.486
	Total	570 (40)	9 (60)	0.119	9 (60)	5 (36)	0.272
Hospitalization, no. (%)	First epidemic	69/126 (55)	2 (100)	0.502	2 (100)	2 (100)	–
	Second epidemic	221/282 (78)	3 (100)	1.000	3 (100)	3 (100)	–
	Third epidemic	178/207 (86)	2 (100)	1.000	2 (100)	2 (100)	–
	Fourth epidemic	84/106 (79)	4 (100)	0.582	4 (100)	3 (100)	–
	Fifth epidemic	636/711 (89)	4 (100)	1.000	4 (100)	4 (100)	–
	Total	1,188/1,432 (83)	15 (100)	0.090	15 (100)	14 (100)	–
ICU admission, no. (%)[a]	First epidemic	36/125 (29)	2 (100)	0.088	2 (100)	2 (100)	–
	Second epidemic	131/282 (46)	3 (100)	0.103	3 (100)	2 (67)	1.000

Characteristics		Comparison of sporadic and cluster-associated index infections			Comparison of cluster-associated index and secondary infections with probable human-to-human transmission		
		Sporadic infections	Index infections	*P* value	Index infections	Secondary infections†	*P* value
Total No. of infections	First epidemic	126	2	–	2	2	–
	Third epidemic	119/207 (57)	2 (100)	0.510	2 (100)	1 (50)	1.000
	Fourth epidemic	58/106 (55)	2 (50)	1.000	2 (50)	1(33)	1.000
	Fifth epidemic	443/693 (64)	4 (100)	0.303	4 (100)	2 (50)	0.429
	Total	787/1,413 (56)	13 (87)	**0.018**	13 (87)	**8 (57)**	0.109
Oseltamivir treatment, no. (%)	First epidemic	51/125 (41)	2 (100)	0.172	2 (100)	2 (100)	–
	Second epidemic	188/282 (67)	2 (67)	1.000	2 (67)	2 (67)	1.000
	Third epidemic	145/207 (70)	1 (50)	0.513	1 (50)	1 (50)	1.000
	Fourth epidemic	67/106 (63)	4 (100)	0.295	4 (100)	2 (67)	0.429
	Fifth epidemic	543/694 (78)	4 (100)	0.582	4 (100)	4 (100)	–
	Total	994/1,414 (70)	13 (87)	0.255	13 (87)	11 (79)	0.651
Mechanical ventilation, no. (%)	First epidemic	28/125 (22)	1 (50)	0.406	1 (50)	1 (50)	1.000
	Second epidemic	97/282 (34)	2 (67)	0.277	2 (67)	1 (33)	1.000
	Third epidemic	94/207 (45)	1 (50)	1.000	1 (50)	0	1.000
	Fourth Epidemic	41/106 (39)	2 (50)	0.643	2 (50)	0	0.429
	Fifth epidemic	310/688 (45)	3 (75)	0.333	3 (75)	0	0.143
	Total	570/1,408 (40)	9 (60)	0.185	9 (60)	2 (14)	**0.021**

*ICU denotes intensive care unit. Categorical variables were compared using Fisher exact test. Median age was compared using rank-sum Wilcoxon test. Boldface indicates significance; –, not applicable.

†Among 3 clusters comprised of 3 case-patients each, two clusters each had one case-patient with probable human-to-human transmission, and in the remaining cluster, the third case-patient had close contact with both of the other 2 case-patients before illness onset, so for this cluster, 2 case-patients are considered index cases in the analyses.

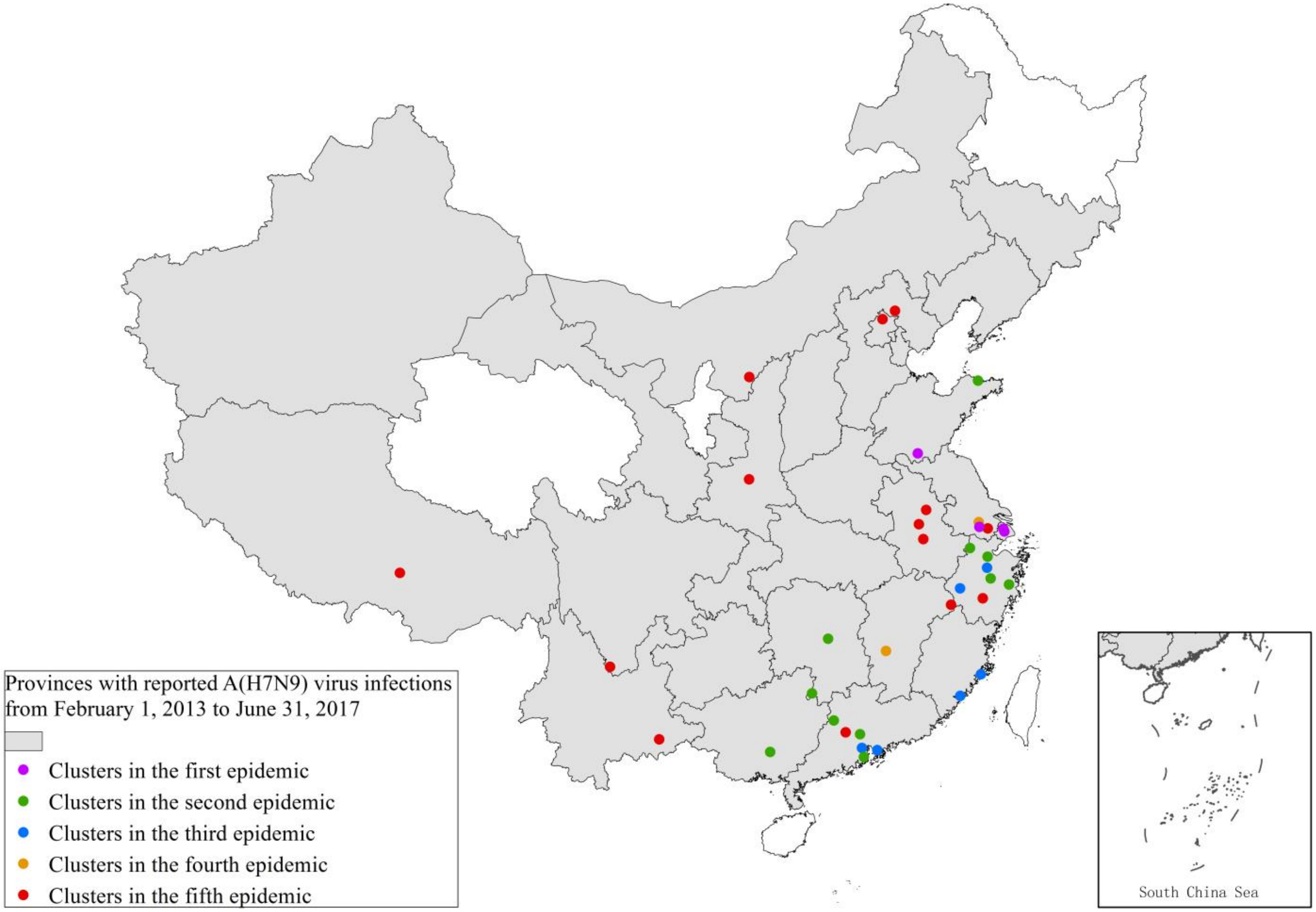

Technical Appendix Figure. Location of clusters of human infections with avian influenza A(H7N9) virus by province and epidemic in Mainland China, February 1, 2013 - June 30, 2017.
